# Receipt of Weight Management Services Among Patients With OSA and Obesity

**DOI:** 10.1016/j.chpulm.2025.100156

**Published:** 2025-03-04

**Authors:** Aristotle G. Leonhard, Jennifer McDowell, Katherine D. Hoerster, Sophia Hayes, Fernando Picazo, Jason M. Castaneda, Kevin Josey, Matthew Griffith, Jun Ma, Kevin I. Duan, Laura C. Feemster, David H. Au, Lucas M. Donovan

**Affiliations:** a Division of Pulmonary, Critical Care, and Sleep Medicine, The University of Washington, Seattle, WA; b Division of Pulmonary, Critical Care, and Sleep Medicine, VA Puget Sound Healthcare System, Seattle, WA; c VA Health Services Research, Seattle Division, VA Puget Sound Healthcare System, Seattle, WA; d Center of Care and Payment Innovation, Office of Healthcare Innovation and Learning, Department of Veterans Affairs, Washington, DC; e Mental Health Services, Seattle Division, VA Puget Sound Healthcare System, Seattle, WA; f Department of Psychiatry and Behavioral Sciences, The University of Washington, Seattle, WA; g Department of Biostatistics & Informatics, Colorado School of Public Health, Aurora, CO; h The Veterans Affairs Eastern Colorado Health Care System, Aurora, CO; i Division of Academic Internal Medicine and Geriatrics, Department of Medicine, The University of Illinois Chicago, Chicago, IL; j Division of Respiratory Medicine, The University of British Columbia, Vancouver, BC, Canada

**Keywords:** lifestyle-based weight management, obesity, OSA, weight loss medications, weight management, health systems

## Abstract

**Background:**

Obesity is the single greatest driver of OSA severity, and clinical practice guidelines recommend weight management services for all patients with OSA and obesity.

**Research Question:**

How often do patients with obesity and newly diagnosed OSA receive weight management services as part of the initial management strategy for OSA? What are the patient- and site-level predictors of receipt of these services?

**Study Design and Methods:**

National electronic health record data from the Veterans Health Administration were used to identify patients with a BMI ≥ 30 kg/m^2^ and a new sleep study diagnostic of OSA. Patients with prior sleep studies, positive airway pressure therapy, or weight management services prior to OSA diagnosis were excluded. The primary study outcome was the receipt of new weight management services in the first 3 to 12 months following diagnosis of OSA. A mixed-effects logistic regression analysis was performed evaluating for patient- and site-level predictors of the receipt of weight management care.

**Results:**

Among 152,976 patients included in our analysis, 15,304 (10.0%) received a weight management service following OSA diagnosis. Of these, 14,146 (9.2%) received a lifestyle-based weight management intervention, 1,790 (1.2%) received a weight management medication, and 29 (0.2%) underwent bariatric surgery. Female sex, Black race, higher BMI, comorbidity burden, and nonrural location were associated with greater receipt of weight management services. The odds of receiving weight management services were also greater among patients cared for at sites that reported greater proportions of patients receiving weight management care in the prior year.

**Interpretation:**

A new OSA diagnosis is an opportunity to consider new treatments. Despite existing guidelines and the availability of services, our results show that patients with OSA and obesity rarely receive weight management care following diagnosis. New strategies are needed to overcome existing barriers to effective weight management care in patients newly diagnosed with OSA.

**Take-Home Points**

**Study Question:** How many patients with obesity and newly diagnosed OSA receive weight management services, and what are the predictors of receipt of these services?

**Results:** This study highlights that 10% of patients with obesity and newly diagnosed OSA receive a weight management service. BMI, sex, race, ethnicity, nonrural location, comorbidities, and site practice patterns predict the receipt of weight management services.

**Interpretation:** Our results indicate that patients with new OSA and obesity rarely receive weight management services. Further work is needed to improve access to weight management care, including in patients with OSA who may derive particular benefits from these services.

Obesity is the single greatest risk factor for the development of OSA, and weight loss is an important part of OSA management.^1^ Numerous randomized trials show that lifestyle-based weight management interventions lead to clinically meaningful weight loss, reductions in OSA severity, and improvements in symptoms.^2^^,^^3^ When lifestyle modification alone is ineffective, additional services for weight management, including pharmacotherapy and bariatric surgery, are known to reduce weight and improve OSA severity.^4^, ^5^, ^6^, ^7^ Given these known benefits, OSA clinical practice guidelines advocate widespread delivery of weight management care, including referrals for comprehensive lifestyle interventions for all patients with OSA and obesity.^8^ In addition, the time of a new diagnosis is perceived to be a window in which patients may be particularly amenable to changes in behavior or new treatments.^9^

Despite growing evidence regarding the benefits of weight management services and the strong recommendations for these interventions in clinical practice guidelines, little is known about the receipt of weight management services among patients with obesity and newly diagnosed OSA. Prior work focused only on the delivery of lifestyle interventions among patients with OSA and found that 8% of patients participated in MOVE!, a nationally implemented behavioral weight management program.^10^^,^^11^ However, little is known about receipt of all available weight management services (eg, MOVE!, nutrition visits, weight loss pharmacotherapy, bariatric surgery). Little is also known about the receipt of these services in the time following a new OSA diagnosis, which may be a window to successfully enact substantial changes in health behaviors. We sought to address this gap by: (1) estimating new delivery of weight management services among patients with obesity and newly diagnosed OSA; and (2) identifying patient- and site-level predictors associated with the receipt of weight management services within this population.

## Study Design and Methods

### Data Source

Nationwide electronic health record data from the Veterans Administration (VA) Corporate Data Warehouse (CDW) were used to conduct this study. The CDW compiles comprehensive data around VA health care delivery, including patient demographic characteristics, vital signs, comorbidities, outpatient encounters, pharmacotherapy, procedures, and locations of treatment. The VA Puget Sound institutional review board (approval #161502) approved this study.

### Population

To create the study cohort, we identified VA patients nationwide with obesity and a new diagnosis of OSA. OSA diagnosis was defined based on documentation in the medical record of the following: (1) a Current Procedural Terminology (CPT) code for either home sleep apnea testing or an in-laboratory sleep study; and (2) an OSA International Classification of Diseases, 10th Revision, diagnostic code. To account for potential variability in the timing of sleep study billing codes relative to study performance, patients with OSA diagnostic codes recorded within 3 months prior to or following documentation of the sleep study CPT code were included (e-Table 1). This creates a 6-month window for an encounter with an OSA diagnosis code to occur (eg, during sleep study interpretation or at a follow-up visit). Patients with sleep study CPT codes documented between October 1, 2017, and December 1, 2021, were identified, and the date of CPT code documentation was used as each patient’s index date.

Given the focus on patients with new diagnoses, we used available data to exclude patients with prior sleep studies or receipt of positive airway pressure (PAP) therapy devices recorded in the VA in the past 5 years or PAP accessories (eg, masks or hoses) recorded in the past 2 years. Obesity was defined based on BMI ≥ 30 kg/m^2^, using the most recent weight recorded in the past year and the most recent height recorded at any time. To restrict the study sample to patients without ongoing weight management care at time of diagnosis, we excluded patients with MOVE! or nutrition encounters in the past year, receipt of weight management medications in the past year, or receipt of bariatric surgery within the past 5 years. Given our priority around understanding the role of geography on service delivery, we excluded patients without available location data. Finally, patients who died within 3 months of the index date were also excluded.

### Outcome

The primary outcome of interest was the receipt of weight management services recorded in CDW, which was defined as: (1) attendance of at least 1 MOVE! or nutrition visit; (2) receipt of a weight management medication (orlistat, phentermine, phentermine-topiramate, bupropion-naltrexone, liraglutide, or semaglutide); or (3) receipt of bariatric surgery. Receipt of a weight management medication is based on a Refill Compliance (ReComp) score greater than 0, which corresponds to at least 1 filled prescription of a weight management medication.^12^ Of note, tirzepatide was not approved by the US Food and Drug Administration until 2022 and therefore was not included in the study cohort. Receipt of bariatric surgery was identified based on corresponding CPT codes (e-Table 1). To ensure outcomes occurred after patients met all inclusion criteria (ie, sleep study plus OSA diagnosis code within 3 months), our primary approach only includes outcomes recorded in CDW at least 3 months following index sleep studies, with follow-up continuing until 12 months. In sensitivity analyses, outcomes recorded in the full 0 to 12 months after index are included.

### Exposures

We identified multiple patient- and site-level characteristics that we hypothesized could affect the delivery of weight management services among veterans with OSA. Incorporating past research,^11^^,^^13^, ^14^, ^15^, ^16^, ^17^ these exposures were selected a priori, their relationships are described in a directed acyclic graph (e-Fig 1).^18^^,^^19^ This directed acyclic graph aligns well with Andersen’s Behavioral Model of Health Services. The Andersen model views access to health care services as being dependent on predisposing characteristics of a population, enabling resources of the population, the need for a service, the health care system/environment, and the personal health practices and health care utilization of the individual.^20^

**Figure 1:**
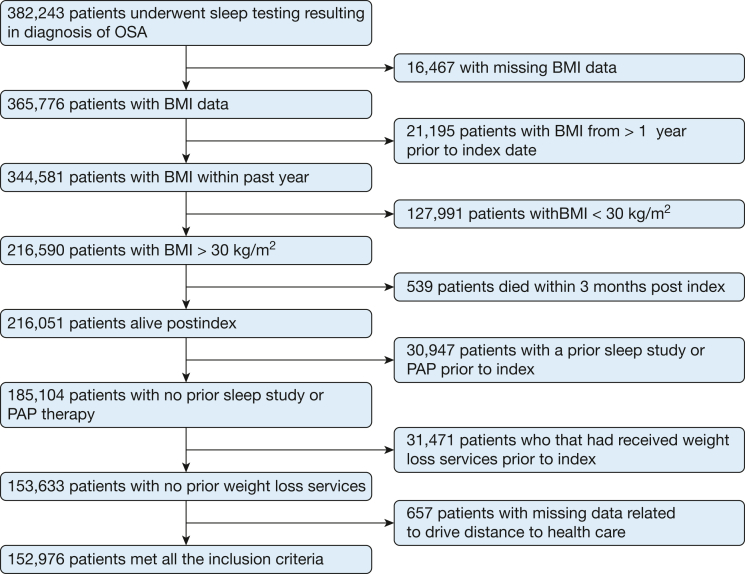
Inclusion/exclusion chart. PAP = positive airway pressure.

#### Patient-Level Exposures

At the patient level, we included demographic characteristics (age, race, ethnicity, sex, and service connection), location (rurality indicator^21^ and drive distance to nearest primary and specialty care), BMI, overall comorbidity burden (Charlson Comorbidity Index score),^22^ individual obesity-related comorbidities (ie, coronary artery disease, type 2 diabetes mellitus, hypertension, and obesity hypoventilation syndrome), and mental health comorbidities (anxiety, depression, and posttraumatic stress disorder).

#### Site-Level Exposures

Prior work suggests that site characteristics play a considerable role in driving the delivery of weight management care.^17^ For this analysis, patient’s site was defined as the location where the patient’s sleep study occurred. At the site level, we included: Complexity Index Designation, volume of patients with OSA, and the proportion of patients with OSA who received weight management care in the year prior to OSA diagnosis. The Complexity Index is based on the Veterans Health Administration (VHA) Facility Complexity Model, which classifies facilities into 5 levels based on the site’s patient population, clinical services complexity, and education/research activities (e-Table 1).^23^ To include volume of patients with OSA cared for at a clinical site, we captured the overall number of patients at each site with newly diagnosed OSA during our study’s time frame. To understand baseline use of weight management care at each clinical site, the proportion of patients with newly diagnosed OSA at each site who had previously received weight management care in the year prior to the sleep study was calculated. Of note, although patients with prior weight management care are used to calculate this site characteristic, these patients were excluded from the analytic sample (as discussed in the Population section).

### Statistical Analysis

We began by describing baseline characteristics of patients who did and did not receive subsequent weight management care. The unadjusted differences between groups were quantified based on standardized mean differences.

The primary analyses were conducted by using a mixed-effects logistic regression model, incorporating patient- and site-level exposures and clustering patients according to site. We determined significance of the various exposures using 2-sided Wald tests of the logit model coefficients. Despite the current model being based on a priori hypotheses, a correction for multiple comparisons testing was still used due to the large number of variables tested in a single model. To minimize alpha inflation and the potential for a type I error, a Bonferroni correction (n = 29) was applied to set a significance level of α = .0017.

Given the changes to health care access and delivery during the COVID-19 pandemic,^24^ a sensitivity analysis was conducted. Specifically, an additional exposure was added to the primary model indicating whether a patient’s index date occurred prior to or following the declaration of the COVID-19 pandemic by the World Health Organization on March 11, 2020.^25^ A second sensitivity analysis was conducted examining the receipt of weight management services 0 to 12 months following the index date, including services received in the first 3 months following sleep study. A third sensitivity analysis was conducted to examine the receipt of weight management services at 3 to 12 months after index stratified according to obesity class (class 1, BMI of 30-34.9 kg/m^2^; class 2, BMI of 35-39.9 kg/m^2^; and class 3, BMI ≥ 40 kg/m^2^). Finally, we conducted a sensitivity analysis stratified according to presence of a diabetes diagnosis, as a cornerstone of diabetes care is weight management.

All analyses were conducted in STATA version 18.0 (StataCorp).

## Results

A total of 382,243 patients were identified who underwent sleep studies with an accompanying diagnosis of OSA. Of these, 229,267 patients were excluded from this analysis for the following factors: missing BMI data (n = 16,467), last BMI > 1 year prior (n = 21,195), BMI < 30 kg/m^2^ (n = 127,991), death < 3 months after index (n = 539), previous sleep study or receipt of PAP prior to index (n = 30,947), receipt of weight management services prior to index (n = 31,471), and missing address data (n = 657). The final cohort comprised 152,976 patients (Fig 1).

The baseline characteristics of the cohort are described in Table 1. The cohort had an average age of 53.1 years (SD, 14.8) and an average BMI of 35.5 kg/m^2^ (SD, 4.6). The cohort was predominantly White (67.3%) and male (89.4%). Most of the individuals lived in urban areas (69.8%) and were diagnosed at a high complexity site (84.8%). The mean Charlson Comorbidity Index score was 1.3 (SD 2.1). Overall, 55.9% of the cohort had hypertension, 24.9% had type 2 diabetes, 13.4% had coronary artery disease, 5.8% had liver disease, and 4.4% had a cerebrovascular accident. Regarding mental health comorbidities, 40.5% of patients had major depressive disorder, 28.8% had posttraumatic stress disorder, and 24.8% had generalized anxiety disorder.

**Table 1:** Baseline Characteristics of the Entire Cohort (N = 152,976)

Characteristic	Value
Patient demographic characteristics	
Age at index, y	53.1 ± 14.8
Female	16,251 (10.6)
Race	
White	102,992 (67.3)
Black	34,528 (22.6)
Native Hawaiian or Pacific Islander	1,748 (1.1)
American Indian or Alaska Native	1,714 (1.1)
Asian	1,481 (1.0)
None provided	10,531 (6.9)
Hispanic ethnicity	15,369 (10.1)
Service connected	112,264 (73.4)
Rurality	
Urban	106,716 (69.7)
Rural	42,918 (28.1)
Highly rural/isolated	3,342 (2.2)
Drive distance to primary care, miles	15.2 ± 13.6
Drive distance to specialty care, miles	39.6 ± 36.4
Medical characteristics	
BMI, kg/m^2^	35.5 ± 4.6
Charlson Comorbidity Index score	1.3 ± 2.1
Comorbidities	
Coronary artery disease	20,446 (13.4)
Type 2 diabetes mellitus	38,109 (24.9)
Obesity hypoventilation syndrome	607 (0.4)
Hypertension	85,435 (55.9)
Posttraumatic stress disorder	44,095 (28.8)
Depression	61,912 (40.5)
Anxiety	37,924 (24.8)
Site characteristics	
Proportion of patients with weight management prior to OSA diagnosis	9.9% ± 3.2
No. of patients with OSA	5,320.3 ± 2901.9
Site complexity	
1a, high complexity	76,754 (50.2)
1b, high complexity	33,130 (21.7)
1c, high complexity	19,827 (13.0)
2, moderate complexity	12,900 (8.4)
3, low complexity	10,067 (6.6)
No complexity data	298 (0.2)

Data are presented as mean ± SD or No. (%).

Among the 152,976 individuals meeting inclusion criteria, 15,304 (10.0%) received weight management services in the 3 to 12 months following the index date. In contrast, 63,962 patients (41.8%) received PAP in the same time frame. Of the 15,304 patients who received a weight management service, the majority (92.4%) received lifestyle-based weight management services, either by attending at least 1 MOVE! session (41.6%) or at least 1 nutrition visit (74.2%). Weight management medications and bariatric surgery were received by a smaller proportion of patients (Table 2). Consistent with the dual role of glucagon-like peptide-1 receptor agonists in weight management and glycemic control, a higher proportion of weight management medication use was observed among those with type 2 diabetes in the study sample (3.4% with type 2 diabetes vs 0.3% without).

**Table 2:** Weight Management Services Received 3 to 12 Months After Index Date

Intervention Received at 12 Months Following New OSA Diagnosis	Entire Cohort (N = 152,976)	With Diabetes (n = 38,109)	Without Diabetes (n = 114,867)
Any weight management intervention	15,304 (10.0)	5,371 (14.1)	9,933 (8.6)
Lifestyle-based weight management intervention	14,146 (9.2)	4,299 (11.3)	9,847 (8.6)
MOVE! visit	6,368 (4.2)	1,472 (3.9)	4,896 (4.3)
Nutrition visit	11,357 (7.4)	3,721 (9.8)	7,636 (6.6)
Prescription for a weight management drug	1,790 (1.2)	1,409 (3.4)	381 (0.3)
Bariatric surgery	29 (< 0.1)	4 (< 0.1)	25 (< 0.1)

Data are presented as No. (%).

**Figure 2:**
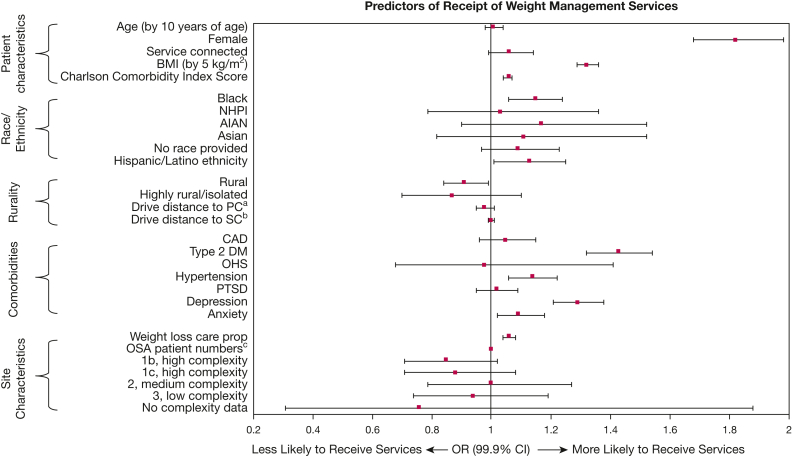
Mixed effects model for predictors of receipt of weight management care. Reference variables in model: race compared with White race and Hispanic compared with non-Hispanic; and rural designation compared with urban. Complexity designation compared with 1a/high complexity. AIAN = American Indian or Alaskan Native; CAD = coronary artery disease; DM = diabetes mellitus; NHPI = Native Hawaiian or Pacific Islander; OHS = obesity hypoventilation syndrome; PTSD = posttraumatic stress disorder; prop = proportion. ^a^Drive distance to specialty care (SC) per 10 miles. ^b^Drive distance to primary care (PC) per 10 miles. ^c^Number of patients with OSA cared for at a specific site.

Table 3 includes unadjusted descriptions of baseline characteristics of patients stratified according to subsequent receipt of weight management services. In a multivariable mixed-effects model (Fig 2, Table 4), several characteristics were associated with receipt of weight management services.

**Table 3:** Standard Differences Based on Whether Weight Management Services Were Received at 3 to 12 Months After Index Date

Variables	Did Not Receive Weight Management Services 3-12 Months After Index Date (n = 137,672)	Received Weight Management Services 3-12 Months After Index Date (n = 15,304)	Standardized Mean Difference
Patient characteristics			
Age at index, y	53.0 ± 14.8	54.4 ± 13.8	0.10
Female	13,677 (9.9)	2574 (16.8)	0.20
Race			0.08
White	93,137 (67.7)	9,855 (64.4)	
Black	30,619 (22.2)	3,909 (25.5)	
Native Hawaiian or Pacific Islander	1,578 (1.1)	170 (1.1)	
American Indian or Alaska Native	1,525 (1.1)	189 (1.2)	
Asian	1,347 (1.0)	134 (0.9)	
None provided	9,466 (6.9)	1,047 (6.8)	
Hispanic ethnicity	13,786 (10.0)	1,583 (10.3)	0.01
Service connected	101,041 (73.4)	11,223 (73.3)	< 0.01
Rurality designation			0.06
Urban	95,643 (69.5)	11,073 (72.4)	
Rural	38,979 (28.3)	3,939 (25.7)	
Highly rural/isolated	3,050 (2.2)	292 (1.9)	
Drive distance to primary care, miles	15.3 ± 13.7	14.4 ± 13.2	0.07
Drive distance to specialty care, miles	39.8 ± 36.4	38.2 ± 35.9	0.04
Medical characteristics			
BMI, kg/m^2^	35.3 ± 4.5	36.7 ± 5.2)	0.29
Charlson Comorbidity Index score	1.2 ± 2.0	1.8 ± 2.4	0.24
Comorbidities			
Coronary artery disease	17,978 (13.1)	2,468 (16.1)	0.09
Type 2 diabetes mellitus	32,738 (23.8)	5,371 (35.1)	0.25
Obesity hypoventilation syndrome	497 (0.4)	110 (0.7)	0.05
Hypertension	75,866 (55.1)	9,569 (62.5)	0.15
Posttraumatic stress disorder	39,495 (28.7)	4,600 (30.1)	0.03
Depression	54,661 (39.7)	7,251 (47.4)	0.16
Anxiety	33,666 (24.5)	4,258 (27.8)	0.08
Site characteristics			
Proportion of patients with weight management prior to OSA diagnosis	9.9% ± 3.1	10.5% ± 3.3	0.21
No. of patients with OSA	5,338 ± 2,909.0	5,157 ± 2,832.2	0.06
Site complexity			0.05
1a, high complexity	68,768 (50.0)	7,986 (52.2)	
1b, high complexity	29,968 (21.8)	3,162 (20.7)	
1c, high complexity	17,950 (13.0)	1,877 (12.3)	
2, moderate complexity	11,643 (8.5)	1,257 (8.2)	
3, low complexity	9,071 (6.6)	996 (6.5)	
No complexity data	272 (0.2)	26 (0.2)	

Data are presented as mean ± SD or No. (%).

**Table 4:** Mixed-Effects Logistic Regression Model for Receipt of Weight Management Services at 3 to 12 Months After Index Date

Characteristic	OR (99.9% CI)
Patient characteristics	
Age at index (per 10 years)	1.01 (0.98-1.04)
Female	**1.82 (1.68-1.98)**
Race (compared with White)	
Black	**1.15 (1.06-1.24)**
Native Hawaiian or Pacific Islander	1.03 (0.79-1.36)
American Indian or Alaska Native	1.17 (0.90-1.52)
Asian	1.11 (0.82-1.52)
None provided	1.09 (0.97-1.23)
Hispanic ethnicity	**1.13 (1.01-1.25)**
Service connected	1.06 (0.99-1.14)
Rurality designation (compared with urban)	
Rural	**0.91 (0.84-0.99)**
Highly rural/isolated	0.88 (0.70-1.10)
Drive distance to primary care (per 10 miles)	0.98 (0.95-1.01)
Drive distance to specialty care (per 10 miles)	1.00 (0.99-1.01)
Medical characteristics	
BMI (per every 5 kg/m^2^)	**1.32 (1.29-1.36)**
Charlson Comorbidity Index score	**1.06 (1.04-1.07)**
Comorbidities	
Coronary artery disease	1.05 (0.96-1.15)
Type 2 diabetes mellitus	**1.43 (1.32-1.54)**
Obesity hypoventilation syndrome	0.98 (0.68-1.41)
Hypertension	**1.14 (1.06-1.22)**
Posttraumatic stress disorder	1.02 (0.95-1.09)
Depression	**1.29 (1.21-1.38)**
Anxiety	**1.09 (1.02-1.18)**
Site characteristics	
Proportion of patients with weight management prior to OSA diagnosis (per 1%)	**1.06 (1.04-1.08)**
No. of patients with OSA (per 100 patients)	1.00 (1.00-1.00)
Site complexity (compared with 1a, high complexity)	
1b, high complexity	0.85 (0.71-1.02)
1c, high complexity	0.88 (0.71-1.08)
2, moderate complexity	1.00 (0.79-1.27)
3, low complexity	0.94 (0.74-1.19)
No complexity data	0.76 (0.31-1.88)

Boldface covariates are significant to a level of *P* < .0017.

Patient characteristics associated with greater receipt of weight management services included higher BMI, greater Charlson Comorbidity Index score, female sex, Black race, and Hispanic or Latino ethnicity. Comorbidities including type 2 diabetes, hypertension, depression, and anxiety also were associated with greater receipt of weight management services. Lower receipt of weight management services was associated with patients living in rural areas.

At the site level, patients were more likely to receive weight management services if their clinical site had provided more weight management services in the last year to patients with OSA.

When we accounted for the COVID-19 pandemic period in a sensitivity analysis, we found that patients were more likely to receive weight management services if their index date occurred following the start of the pandemic. None of the other associations was substantially altered by the addition of this consideration into the mixed-effects model (e-Table 2).

Sensitivity analyses were also conducted in which we included outcomes from 0-12 months following sleep study, which includes the 3-month period in which some patients may not yet have had an OSA diagnosis code documented in the electronic health record. Within this entire 12-month period, we found that 12% of patients receive weight management care, relative to 75% who received PAP therapy. Using this 12-month outcome period, predictors of weight management care were comparable to the primary analysis (e-Table 3).

Finally, sensitivity analyses were conducted assessing the receipt of weight management services stratified according to obesity class and by the presence of a diabetes diagnosis. Overall, the general trend and magnitude of relationships remained unchanged based on obesity class (e-Table 4). When analyses were stratified based on the presence or absence of diabetes, qualitative differences were observed between strata in the magnitude and direction of some observed associations. Notably, age was positively associated with weight management receipt among those without diabetes and negatively associated among those with diabetes. In addition, Black race was associated with receipt of weight management care among those without comorbid diabetes, but no clear association was observed among those with diabetes (e-Table 5).

## Discussion

Among VA patients with obesity and newly diagnosed OSA, we found that patients rarely receive weight management services within the first 3 to 12 months following diagnosis of OSA. Even when defining the outcome of receipt of a weight loss intervention broadly to include lifestyle programs, medications, and surgery to capture the range of strategies that can be offered by the health system, only 1 in 10 patients received any of these formal interventions. The establishment of a new diagnosis is perceived by many to be an opportunity to help patients alter health behaviors and initiate new treatments.^9^ Given the role of excess weight in driving the development and severity of OSA,^1^ weight management therapies are particularly important following a new OSA diagnosis. However, despite decades of research and health system investments, a large gap remains between guideline-based recommendations and the delivery of weight management care to patients with OSA.^11^ The current findings also suggest multiple drivers that affect likelihood of receipt of these services, including multimorbidity, sex, race, rurality, and site of care.

Our findings reinforce the role of comorbidity burden as a key predictor of weight management care receipt. We found that both overall comorbidity burden (Charlson Comorbidity Index score) and specific obesity-related comorbidities were independently associated with the receipt of weight management services. As described in Andersen’s Behavioral Model of Health Services, perceived need for a given service is a key motivator for health care use.^20^ In the setting of multimorbidity, patients may derive substantial benefits from weight management, and anticipation of these benefits may motivate patients to seek care and providers to recommend these interventions.^26^^,^^27^ In addition, the specific comorbidities of type 2 diabetes, hypertension, anxiety, and depression were all independently associated with a higher receipt of weight management services. Weight management is unambiguously established in guidelines as part of the multimodal treatment for type 2 diabetes^28^ and hypertension,^29^ providing clear indications for referral among these patients. There are also potential benefits of weight loss for mental health disorders such as depression and anxiety.^30^ Notably, we also found that that a diagnosis of diabetes may modify the relationship between patient characteristics and weight management receipt. These results reinforce the need to consider comorbid diabetes in future efforts aimed at understanding and improving the receipt of weight management care in OSA. Beyond medical conditions, weight management may also improve mental health disorders,^31^^,^^32^ and we observed an association between anxiety and depression diagnoses with delivery of weight management services.

We found that patients of female sex were substantially more likely to receive weight management services. These findings align with previous work showing that women are more likely to receive weight management counseling, pharmacotherapy, and bariatric surgery.^11^^,^^15^^,^^33^ In turn, these observed differences reflect known sex- and gender-based differences in weight perception and stigma.^34^ In light of these findings, it is important that we consider ways to approach recommendations around weight management care among patients with OSA in a way that reaches across genders and avoids sex- and gender-based weight stigma.

Similar to prior work in the VA’s MOVE! program, we also found that patients identifying as Black were more likely to receive weight management services.^11^ These findings contrast to work outside of the VA that found lower receipt of weight management services in this population.^14^ The mechanisms of these disparate findings are unclear but may reflect geographic clustering of centers offering MOVE!, differential drivers of access across health systems, and biases around weight management referrals. In contextualizing these results, it is important to note also that patients identifying as Black who participate in a lifestyle-based weight management program tend to lose less weight relative to their peers who identify as White.^35^^,^^36^ Particularly given known disparities in OSA treatments, it is important that we better understand this gap in equity and pursue strategies to overcome it.

Our work also highlights disparities for rural patients, who were less likely to receive weight management care. Among patients in rural areas, the most obvious access barriers are geographic.^37^ Accordingly, telehealth options are seen as a particularly attractive option for weight management services, extending the reach of such services as lifestyle programs (eg, TeleMOVE!) and counseling around weight management pharmacotherapy. In our sensitivity analyses, patients were more likely to receive a weight management service if their index date occurred following the start of the COVID-19 pandemic. It is possible that rapid adoption of telehealth options, including for MOVE!, during the pandemic at least partially explains our observations around greater receipt of weight management services during this period.^24^ This finding also highlights that telehealth may overcome substantial challenges to, and improve, the delivery of weight management services. However, barriers to access among rural patients transcend geography. For instance, patients in rural areas may also have limited access to telehealth services due to poor broadband access.^37^ Given overlapping risk factors for obesity and comorbid conditions among patients in rural areas, more work will be needed to effectively deliver weight management care to these patients.^38^^,^^39^

Similar to prior work, the current findings also reinforce the important role that health systems play in delivering weight management services.^11^^,^^17^ Accounting for all other characteristics, patients were more likely to receive weight management services if they were cared for at a site that more often delivered these services. Effective weight management care requires substantial health system investments and coordination across a range of disciplines, including experts in behavioral health, endocrinology, and bariatric surgery. Recent efforts have been made to enhance coordination between these experts and primary care providers.^40^ However, as the vast majority of patients receive OSA services in specialized sleep centers,^41^ we need to ensure integration of weight management services with sleep centers as well.

Meanwhile, we also need to consider the relative effectiveness of our various options.^42^ For instance, intensive lifestyle interventions have been shown to lead to clinically meaningful weight loss in randomized trials,^43^ but the real-world effectiveness of these programs is often less robust.^10^ Beyond lifestyle interventions, alternative options are available that meaningfully augment weight loss, including pharmacotherapy^5^^,^^6^ and bariatric surgery.^7^ Unfortunately, our results show that these highly efficacious alternative options are rarely used. Future work will need to better understand the optimal integration of these services and assess their real-world effectiveness in generalizable settings.

A major strength of the current study is the setting. The VHA is the nation’s largest vertically integrated health system,^44^ with a comprehensive electronic medical record database. By conducting our study in the VHA, we are able to understand associations between weight management care with important patient characteristics (eg, measured BMI, distance from care, comorbidities) that are frequently absent or have substantial missingness in administrative data sets (eg, Medicare).^45^ Patients within the VA are also able to access lifestyle weight management services (eg, MOVE!) free of charge nationwide, relieving a major financial barrier to care.

The current study also has some limitations. Although the use of administrative and electronic health record data enables analyses of generalizable populations, there are risks for misclassification.^46^ For instance, we included nutrition visits in our composite outcome of weight management care. Although a diet focused on a healthy weight is a key component of nutritional counseling, it is possible that weight management was not addressed at some of these nutrition visits.^47^ In addition, our VA data set did not include exposures that may affect referrals for weight management care, such as OSA severity (ie, apnea hypopnea index) or OSA symptoms. Several aspects of our cohort may affect generalizability and misclassification. First, we included patients with an OSA International Classification of Diseases, 10th Revision, code within 3 months (either prior to or following) a sleep study CPT code. This approach may have omitted some patients with less timely OSA management. This approach may also have included patients who did not have OSA identified on their sleep study; this is unlikely, however, given the very low rate of negative sleep studies in the VHA and the high rate of subsequent PAP receipt in the current cohort (75%).^48^ Patients without OSA and patients with less timely assessment of OSA may be less likely to receive weight management services. Future work should consider varying definitions of OSA using administrative data, and the impact of delayed OSA treatment on weight management receipt and patient outcomes. Second, we designed our study to assess outcomes among those with BMI in the obese range (BMI ≥ 30 kg/m^2^). However, weight management services are also recommended for patients with OSA in the overweight category (BMI 25-29.9 kg/m^2^), and future work should consider this group.^8^ Third, although our cohort includes a large number of female individuals (16,251 patients) and patients from underrespresented groups (39,471 patients), they comprise a lower proportion in the study cohort than in the general population.^49^ Finally, our data set is also limited to VA-provided services. Although there is a strong cost incentive for patients to use VA care, some patients may seek weight management care outside of the VA system, and these services would not be captured. Given the nature of the Veterans Health Administration, patients can access services such as MOVE! without co-pay or referral.^50^ It is therefore possible that our findings may not generalize to populations served outside of the VA.

## Interpretation

A new diagnosis represents an opportunity to consider new therapies. Despite the recommendations of evidence-based guidelines, the receipt of weight management services among patients with obesity and newly diagnosed OSA remains low. Our findings underscore that additional work is needed to improve access to weight management care across our population. As we do so, we need to promote equitable delivery across racial groups, sexes, geographic regions, and health systems.

## Funding/Support

A. G. L. received support during the period of this work from a 10.13039/100000002National Institutes of Health T32 training grant through the 10.13039/100007812University of Washington Division of Pulmonary, Critical Care, and Sleep Medicine [NIH 5T32HL007287-45]. L. M. D. received support during the period of this work from VA 10.13039/100007217Health Services Research CDA 18-187.

## Financial/Nonfinancial Disclosures

The authors have reported to *CHEST Pulmonary* the following: L. M. D. reports an honorarium from the American Academy of Sleep Medicine for participation in a postgraduate course. D. H. A. reports personal fees from Boehringer Ingelheim for service on an advisory board and personal fees from the American Thoracic Society for service as a deputy editor. L. C. F. reports personal fees from the *Annals of the American Thoracic Society* for service as associate editor; and receives consulting fees from the National Committee for Quality Assurance for curriculum development and the Society for Hospital Medicine and American Thoracic Society to serve on expert panels. None declared (A. G. L., J. M., K. D. H., S. H., F. P., J. M. C., K. J., M. G., J. M., K. I. D.).

## Supplementary Data

e-Online Data
