# Supplementary material for: Receipt of Weight Management Services Among Patients With OSA and Obesity
Source: CHEST Pulm. 2025 Mar 4;3(2):100156. doi: 10.1016/j.chpulm.2025.100156 (PMC13193224; doi:10.1016/j.chpulm.2025.100156)
Supplement: e-Online Data [file mmc1.docx]

Supplemental information:

e-Table 1: Codes to identify sleep studies, diagnoses, PAP supplies, weight management medications, and site complexity

| **Sleep Study CPT codes** | |
| --- | --- |
| 95800 | Sleep study, unattended |
| 95801 | Sleep study, unattended |
| 95806 | Sleep study, unattended |
| 95810 | Polysomnogram, in lab |
| 95811 | Polysomnogram, in lab, split night |
| **OSA diagnosis codes** | |
| G47.30 | Sleep apnea, unspecified |
| G47.33 | Obstructive sleep apnea |
| **PAP supplies HCPS codes** | |
| E0601 | CPAP device |
| E0450 | Volume control ventilator |
| E0454 | Pressure ventilator |
| E0461 | Volume control noninvasive ventilator |
| E0463 | Pressure support ventilator |
| E0464 | Pressure support noninvasive ventilator |
| E0465 | Home ventilator |
| E0466 | Noninvasive home ventilator |
| E0467 | Home ventilator, multi function |
| E0470 | Bilevel PAP without a backup rate |
| E0471 | Bilevel Pap with a backup rate |
| E0561 | Humidifier, nonheated, for PAP |
| E0562 | Humidifier, heated, for PAP |
| A4604 | Heated tubing |
| A7027 | Hybrid mask |
| A7028 | Replacement hybrid mask |
| A7029 | Replacement hybrid mask nasal pillow |
| A7030 | CPAP full face mask |
| A7031 | Replacement facemask interface |
| A7032 | Replacement nasal cushion |
| A7033 | Replacement nasal pillows |
| A7034 | Nasal application device |
| A7035 | PAP headgear |
| A7036 | PAP chinstrap |
| A7037 | PAP tubing |
| A7038 | PAP filter |
| A7039 | PAP filter, nondisposable |
| A7045 | Replacement exhalation port for PAP |
| A7046 | Replacement water chamber for PAP device. |
| **Bariatric Procedure codes** |  |
| 43644 | LAPAROSCOPIC GASTRIC BYPASS/ROUX-EN-Y |
| 43773 | LAPAROSCOPIC REPLACE GASTR ADJ DEVICE |
| 43842 | V-BAND GASTROPLASTY |
| 43843 | GASTROPLASTY W/O V-BAND |
| 43846 | GASTRIC BYPASS FOR OBESITY |
| 43847 | GASTRIC BYPASS INCL SMALL I |
| 43848 | REVISION GASTROPLASTY |
| 43999 | UNLISTED PROCEDURE STOMACH |
| 43770 | LAP PLACE GASTR ADJ DEVICE |
| 43772 | LAP RMVL GASTR ADJ DEVICE |
| 43888 | CHANGE GASTRIC PORT OPEN |
| 43645 | LAP GASTR BYPASS INCL SMLL I |
| 43774 | LAP RMVL GASTR ADJ ALL PARTS |
| 43775 | LAP SLEEVE GASTRECTOMY |
| 43886 | REVISE GASTRIC PORT OPEN |
| 43771 | LAP REVISE GASTR ADJ DEVICE |
| 43845 | GASTROPLASTY DUODENAL SWITCH |
| 43887 | REMOVE GASTRIC PORT OPEN |
| **Weight management medications** | |
| Orlistat | |
| Semaglutide | |
| Liraglutide | |
| Bupropion-Naltrexone | |
| Phentermine-Topiramate | |
| Phentermine | |
| **Clinical Complexity Index Designations** | |
| 1a-Highest complexity | Facilities with high volume, high risk patients, most complex clinical programs, and large research and teaching programs |
| 1b-Highest complexity | Facilities with medium-high volume, high risk patients, many complex clinical programs, and medium-large research and teaching programs |
| 1c-Mid-High complexity | Facilities with medium-high volume, medium risk patients, some complex clinical programs, and medium sized research and teaching programs |
| 2-Medium complexity | Facilities with medium volume, low risk patients, few complex clinical programs, and small or no research and teaching programs |
| 3-Low complexity | Facilities with low volume, low risk patients, few or no complex clinical programs, and small or no research and teaching programs |

Legend: CPAP- continuous positive airway pressure; PAP- positive airway pressure

e-Figure 1: Directed acyclic graph of factors associated with receipt of weight management care


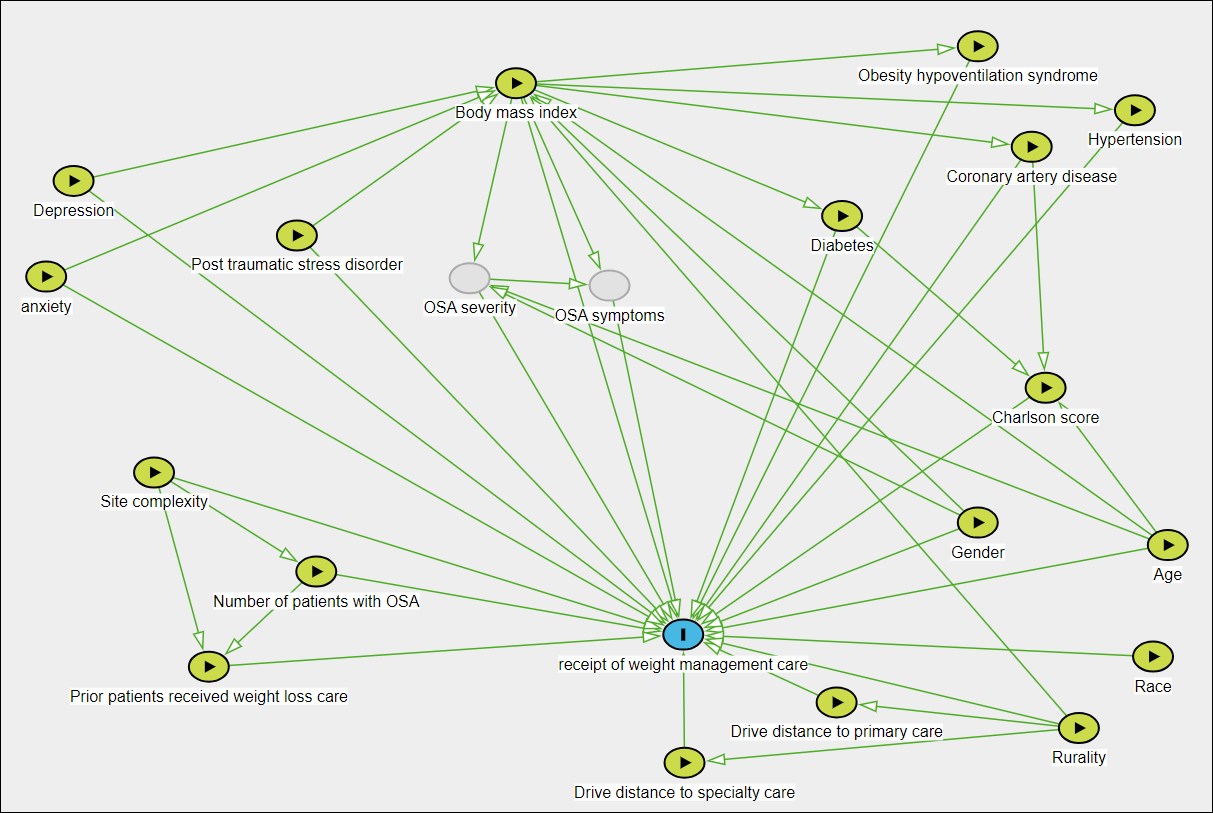


Textor J, Van Der Zander B, Gilthorpe MS, Liśkiewicz M, Ellison GTH. Robust causal inference using directed acyclic graphs: the R package ‘dagitty.’ Int J Epidemiol. Published online January 15, 2017:dyw341. doi:10.1093/ije/dyw341

Legend: OSA-obstructive sleep apnea.

e-Table 2: Sensitivity analysis with mixed effects logistic regression model for receipt of weight management services 3-12 months after index, taking into account if index date was before or after start of the COVID pandemic

|  | OR (99.9%CI) |
| --- | --- |
| *Patient Characteristics* | |
| Age at index (per 10 years) | 1.01 (0.98-1.04) |
| **Female** | **1.80 (1.66-1.96)** |
| Race (as compared to Black race) |  |
| **Black** | **1.15 (1.07-1.24)** |
| Native Hawaiian or Pacific Islander | 1.03 (0.78-1.35) |
| American Indian or Alaska Native | 1.17 (0.90-1.52) |
| Asian | 1.12 (0.82-1.52) |
| None provided | 1.08 (0.96-1.21) |
| **Hispanic ethnicity** | **1.13 (1.01-1.26)** |
| Service connected | 1.05 (0.99-1.13) |
| Rurality designation (as compared to urban) |  |
| **Rural** | **0.91 (0.84-0.99)** |
| Highly rural/Isolated | 0.85 (0.68-1.07) |
| Drive distance to primary care (per 10 miles) | 0.98 (0.95-1.01) |
| Drive distance to specialty care (per 10 miles) | 1.00 (0.99-1.01) |
| **Index date occurred after start of COVID pandemic** | **1.32 (1.24-1.41)** |
| *Medical Characteristics* | |
| **Body mass index (per every 5 kg/m^2^)** | **1.32 (1.29-1.36)** |
| **Charlson score** | **1.06 (1.04-1.07)** |
| Comorbidities |  |
| Coronary artery disease | 1.05 (0.97-1.15) |
| **Type 2 diabetes mellitus** | **1.44 (1.33-1.55)** |
| Obesity hypoventilation syndrome | 0.98 (0.68-1.41) |
| **Hypertension** | **1.14 (1.06-1.22)** |
| Post traumatic stress disorder | 1.02 (0.95-1.09) |
| **Depression** | **1.29 (1.21-1.38)** |
| **Anxiety** | **1.08 (1.01-1.16)** |
| *Site Characteristics* | |
| **Proportion of patients with weight management prior to OSA diagnosis (per 1%)** | **1.06 (1.04-1.08)** |
| Number of patients with OSA (per 100 patients) | 1.00 (1.00-1.00) |
| Site complexity (as compared to 1a-high complexity) |  |
| 1b-high complexity | 0.84 (0.70-1.02) |
| 1c- high complexity | 0.88 (0.71-1.09) |
| 2- moderate complexity | 1.00 (0.78-1.28) |
| 3- low complexity | 0.94 (0.74-1.20) |
| No complexity data | 0.80 (0.32-2.01) |

*Bolded covariates were significant to a level of *P*<0.0017

Legend: OR- Odds ratio; CI- Confidence interval; OSA-Obstructive sleep apnea.

e-Table 3: Sensitivity analysis with a mixed effects logistic regression model for receipt of weight management services at 0-12 months post index

|  | OR (99.9%CI) |
| --- | --- |
| *Patient Characteristics* | |
| Age at index (per 10 years) | 0.99 (0.96-1.01) |
| **Female** | **1.76 (1.63-1.90)** |
| Race (as compared to white) |  |
| **Black** | **1.18 (1.10-1.25)** |
| Native Hawaiian or Pacific Islander | 1.14 (0.89-1.45) |
| American Indian or Alaska Native | 1.17 (0.92-1.48) |
| Asian | 1.14 (0.87-1.51) |
| **None provided** | **1.12 (1.01-1.25)** |
| **Hispanic ethnicity** | **1.14 (1.03-1.26)** |
| Service connected | 1.02 (0.96-1.09) |
| Rurality designation (as compared to urban) |  |
| **Rural** | **0.91 (0.84-0.98)** |
| Highly rural/Isolated | 0.84 (0.68-1.04) |
| Drive distance to primary care (per 10 miles) | 0.98 (0.96-1.01) |
| Drive distance to specialty care (per 10 miles) | 1.00 (0.99-1.00) |
| *Medical Characteristics* | |
| **Body mass index (per every 5 kg/m^2^)** | **1.33 (1.30-1.37)** |
| **Charlson score** | **1.06 (1.04-1.07)** |
| Comorbidities |  |
| Coronary artery disease | 1.06 (0.97-1.15) |
| **Type 2 diabetes mellitus** | **1.39 (1.30-1.49)** |
| Obesity hypoventilation syndrome | 1.01 (0.72-1.42) |
| **Hypertension** | **1.14 (1.07-1.21)** |
| Post traumatic stress disorder | 1.02 (0.96-1.09) |
| **Depression** | **1.28 (1.20-1.36)** |
| **Anxiety** | **1.11 (1.04-1.18)** |
| *Site Characteristics* | |
| **Proportion of patients with weight management prior to OSA diagnosis (per 1%)** | **1.06 (1.04-1.08)** |
| Number of patients with OSA (per 100 patients) | 1.00 (1.00-1.00) |
| Site complexity (as compared to 1a-high complexity) |  |
| 1b-high complexity | 0.86 (0.71-1.03) |
| 1c- high complexity | 0.90 (0.73-1.11) |
| 2- moderate complexity | 1.01 (0.80-1.29) |
| 3- low complexity | 0.96 (0.76-1.22) |
| No complexity data | 0.78 (0.32-1.89) |

*Bolded covariates were significant to a level of *P*<0.0017

Legend: OR- Odds ratio; CI- Confidence interval; OSA-Obstructive sleep apnea.

e-Table 4: Sensitivity analysis with a mixed effects logistic regression model for receipt of weight management services at 3-12 months post index stratified by obesity class

|  | BMI 30-34.9  N=85,725  OR (99.9%CI) | BMI 35-39.9  N=44,944  OR (99.9%CI) | BMI $\geq40$  N= 22,307  OR (99.9%CI) |
| --- | --- | --- | --- |
| *Patient Characteristics* | | | |
| Age at index (per 10 years) | 1.00 (0.96-1.04) | 1.03 (0.99-1.08) | 1.00 (0.94-1.06) |
| Female | **2.03 (1.80-2.30)** | **1.86 (1.61-2.14)** | **1.47 (1.23-1.74)** |
| Race (as compared to white) | | | |
| Black | **1.14 (1.02-1.27)** | 1.14 (1.00-1.29) | 1.14 (0.97-1.34) |
| Native Hawaiian or Pacific Islander | 1.28 (0.88-1.85) | 0.75 (0.44-1.28) | 0.91 (0.49-1.69) |
| American Indian or Alaska Native | 1.11 (0.74-1.65) | 1.38 (0.89-2.14) | 1.06 (0.60-1.85) |
| Asian | 1.14 (0.77-1.68) | 0.95 (0.51-1.76) | 1.29 (0.53-3.16) |
| None provided | **1.03 (0.87-1.23)** | 1.15 (0.94-1.40) | 1.13 (0.88-1.45) |
| Hispanic ethnicity | 1.14 (0.98-1.33) | 1.11 (0.93-1.34) | 1.09 (0.87-1.37) |
| Service connected | 1.02 (0.92-1.13) | 1.12 (1.00-1.27) | 1.02 (0.89-1.18) |
| Rurality designation (as compared to urban) | | | |
| Rural | **0.88 (0.78-0.99)** | 0.94 (0.82-1.08) | 0.94 (0.79-1.12) |
| Highly rural/Isolated | 0.78 (0.55-1.10) | 0.84 (0.56-1.27) | 1.14 (0.73-1.79) |
| Drive distance to primary care (per 10 miles) | 0.99 (0.95-1.03) | 0.97 (0.92-1.01 | 0.98 (0.93-1.04) |
| Drive distance to specialty care (per 10 miles) | 1.00 (0.98-1.01) | 1.00 (0.98-1.02) | 1.00 (0.98-1.02) |
| *Medical Characteristics* | | | |
| Charlson score | **1.06 (1.04-1.09)** | **1.05 (1.02-1.08)** | **1.04 (1.01-1.08)** |
| Comorbidities | | | |
| Coronary artery disease | 1.05 (0.92-1.19) | 1.00 (0.86-1.17) | 1.08 (0.89-1.32) |
| Type 2 diabetes mellitus | **1.58 (1.41-1.76)** | **1.39 (1.22-1.58)** | **1.27 (1.08-1.49)** |
| Obesity hypoventilation syndrome | 0.84 (0.20-3.59) | 0.86 (0.38-1.95) | 1.31 (0.86-1.99) |
| Hypertension | **1.20 (1.08-1.33)** | 1.09 (0.97-1.24) | 1.10 (0.94-1.28) |
| Post traumatic stress disorder | 1.02 (0.92-1.12) | 1.02 (0.91-1.15) | 1.01 (0.87-1.18) |
| Depression | **1.31 (1.19-1.44)** | **1.23 (1.09-1.37)** | **1.36 (1.18-1.57)** |
| Anxiety | **1.11 (1.01-1.23)** | 1.08 (0.95-1.22) | 1.07 (0.91-1.25) |
| *Site Characteristics* | | | |
| Proportion of patients with weight management prior to OSA diagnosis (per 1%) | **1.06 (1.04-1.09)** | **1.07 (1.05-1.09)** | **1.06 (1.03-1.09)** |
| Number of patients with OSA (per 100 patients) | 1.00 (0.99-1.00) | 1.00 (1.00-1.00) | 1.00 (0.99-1.00) |
| Site complexity (as compared to 1a-high complexity) | | | |
| 1b-high complexity | 0.85 (0.69-1.05) | 0.91 (0.75-1.10) | 0.83 (0.66-1.05) |
| 1c- high complexity | 0.85 (0.66-1.09) | 0.91 (0.72-1.14) | 0.91 (0.68-1.20) |
| 2- moderate complexity | 0.98 (0.73-1.31) | 1.06 (0.81-1.39) | 0.97 (0.70-1.35) |
| 3- low complexity | 0.89 (0.66-1.20) | 0.98 (0.73-1.30) | 0.99 (0.70-1.41) |
| No complexity data | 0.71 (0.21-2.42) | 0.95 (0.28-3.24 | 0.60 (0.45-1.32 |

*Bolded covariates were significant to a level of *P*<0.0017

Legend: OR- Odds ratio; CI- Confidence interval; OSA-Obstructive sleep apnea.

e-Table 5: Sensitivity analysis with a mixed effects logistic regression model for receipt of weight management services at 3-12 months post index stratified by presence of diabetes diagnosis

|  | With Diabetes  N=38,109  OR (99.9%CI) | Without Diabetes  N=114,867  OR (99.9%CI) |
| --- | --- | --- |
| *Patient Characteristics* | | |
| Age at index (per 10 years) | **0.89 (0.85-0.95)** | **1.05 (1.02-1.08)** |
| Female | **1.47 (1.23-1.76)** | **1.90 (1.73-2.09)** |
| Race (as compared to white) |  |  |
| Black | 0.98 (0.86-1.35) | **1.23 (1.12-1.35)** |
| Native Hawaiian or Pacific Islander | 0.99 (0.62-1.58) | 1.04 (0.75-1.46) |
| American Indian or Alaska Native | 0.76 (0.45-1.29) | 1.40 (1.03-1.89) |
| Asian | 1.37 (0.81-2.33) | 1.00 (0.68-1.47) |
| None provided | 1.09 (0.88-1.35) | 1.09 (0.95-1.25) |
| Hispanic ethnicity | 1.07 (0.88-1.30) | **1.15 (1.01-1.30)** |
| Service connected | 1.06 (0.95-1.18) | 1.07 (0.98-1.17) |
| Rurality designation (as compared to urban) |  |  |
| Rural | 0.88 (0.76-1.01) | 0.93 (0.84-1.03) |
| Highly rural/Isolated | 0.81 (0.56-1.18) | 0.90 (0.68-1.21) |
| Drive distance to primary care (per 10 miles) | 0.98 (0.94-1.03) | 0.98 (0.95-1.01) |
| Drive distance to specialty care (per 10 miles) | 1.01 (0.99-1.02) | 0.99 (0.98-1.01) |
| *Medical Characteristics* | | |
| Body mass index (per every 5 kg/m^2^) | **1.04 (1.03-1.05)** | **1.39 (1.34-1.43)** |
| Charlson score | **1.07 (1.05-1.09)** | **1.06 (1.03-1.08)** |
| Comorbidities |  |  |
| Coronary artery disease | 1.05 (0.93-1.19) | 1.05 (0.93-1.20) |
| Obesity hypoventilation syndrome | 1.06 (0.67-1.69) | 1.02 (0.56-1.84) |
| Hypertension | **1.18 (1.02-1.37)** | **1.10 (1.01-1.19)** |
| Post traumatic stress disorder | 0.97 (0.86-1.11) | 1.04 (0.96-1.13) |
| Depression | **1.27 (1.14-1.43)** | **1.30 (1.20-1.40)** |
| Anxiety | 1.01 (0.89-1.16) | **1.10 (1.01-1.19)** |
| *Site Characteristics* | | |
| Proportion of patients with weight management prior to OSA diagnosis (per 1%) | **1.05 (1.03-1.08)** | **1.07 (1.05-1.09)** |
| Number of patients with OSA (per 100 patients) | 1.00 (0.99-1.00) | 1.00 (1.00-1.00) |
| Site complexity (as compared to 1a-high complexity) |  |  |
| 1b-high complexity | 0.86 (0.69-1.06) | 0.85 (0.70-1.05) |
| 1c- high complexity | 0.88 (0.68-1.12) | 0.88 (0.69-1.11) |
| 2- moderate complexity | 0.99 (0.74-1.33) | 1.00 (0.76-1.31) |
| 3- low complexity | 0.99 (0.73-1.34) | 0.92 (0.70-1.20) |
| No complexity data | 0.78 (0.21-2.93) | 0.76 (0.26-2.19) |
